# Divergent Effects of Metformin on an Inflammatory Model of Parkinson’s Disease

**DOI:** 10.3389/fncel.2018.00440

**Published:** 2018-11-21

**Authors:** Khadija Tayara, Ana M. Espinosa-Oliva, Irene García-Domínguez, Afrah Abdul Ismaiel, Antonio Boza-Serrano, Tomas Deierborg, Alberto Machado, Antonio J. Herrera, José L. Venero, Rocío M. de Pablos

**Affiliations:** ^1^Instituto de Biomedicina de Sevilla (IBiS), Hospital Universitario Virgen del Rocío/CSIC/Universidad de Sevilla, Seville, Spain; ^2^Departamento de Bioquímica y Biología Molecular, Facultad de Farmacia, Universidad de Sevilla, Seville, Spain; ^3^Experimental Neuroinflammation Laboratory, Department of Experimental Medical Science, Lund University, Lund, Sweden

**Keywords:** neuroinflammation, Parkinson’s disease, metformin, microglia activation, animal model, AMPK

## Abstract

The oral antidiabetic drug metformin is known to exhibit anti-inflammatory properties through activation of AMP kinase, thus protecting various brain tissues as cortical neurons, for example. However, the effect of metformin on the substantia nigra (SN), the main structure affected in Parkinson’s disease (PD), has not yet been studied in depth. Inflammation is a key feature of PD and it may play a central role in the neurodegeneration that takes place in this disorder. The aim of this work was to determine the effect of metformin on the microglial activation of the SN of rats using the animal model of PD based on the injection of the pro-inflammogen lipopolysaccharide (LPS). *In vivo* and *in vitro* experiments were conducted to study the activation of microglia at both the cellular and molecular levels. Our results indicate that metformin overall inhibits microglia activation measured by OX-6 (MHCII marker), IKKβ (pro-inflammatory marker) and arginase (anti-inflammatory marker) immunoreactivity. In addition, qPCR experiments reveal that metformin treatment minimizes the expression levels of several pro- and anti-inflammatory cytokines. Mechanistically, the drug decreases the phosphorylated forms of mitogen-activated protein kinases (MAPKs) as well as ROS generation through the inhibition of the NADPH oxidase enzyme. However, metformin treatment fails to protect the dopaminergic neurons of SN in response to intranigral LPS. These findings suggest that metformin could have both beneficial and harmful pharmacological effects and raise the question about the potential use of metformin for the prevention and treatment of PD.

## Introduction

Parkinson’s disease (PD) is a neurodegenerative disorder featuring the loss of the dopaminergic neurons in the SN and Lewy pathology (intracellular accumulation of α-synuclein and other proteins in the so-called Lewy bodies), with a resultant alteration of the motor function that produces the loss of autonomy; cognitive function and mood are also impaired (Marinus et al., 2018; Odin et al., 2018; Sojitra et al., 2018). The disease occurs in two forms with different causes and prevalence, so that the so-called early onset family form is due to mutations in a few genes and represent no more than 10% of cases, while the most frequent form, associated with aging (the idiopathic or sporadic form, typically occurring around 65 years of age), shows a diffuse etiology. Several factors affecting common processes in cells, such as the loss of mitochondrial function (and the associated oxidative stress) induced or not by toxins, the alteration of proteostatic processes (including autophagy and the ubiquitin-proteasome pathway) or the neuroinflammatory processes associated or not with infections, have been found to contribute to the onset and development of sporadic cases (Herrera et al., 2015; De Miranda et al., 2018; Huang H.K. et al., 2018; Scudamore and Ciossek, 2018; Zeng et al., 2018).

The early report by McGeer et al. (1988) showing the presence of microglia in parkinsonian brains has been followed by others that confirm the existence of a neuroinflammatory process underlying many cases of the disease (Klegeris et al., 2007; McGeer and McGeer, 2008). In addition, activated microglia show increased production of pro-inflammatory cytokines (Tansey et al., 2007; Hirsch and Hunot, 2009). One point in favor of the role of neuroinflammation in PD is the lower incidence (around 50%) of the disease in chronic users of non-steroidal anti-inflammatory drugs and cyclooxygenase inhibitors (Chen et al., 2003, 2005; Esposito et al., 2007). From this observation, many studies have attempted to prove the capacity of different substances with anti-inflammatory properties to reduce the progression of PD (Cayero-Otero et al., 2018). These substances include non-steroidal anti-inflammatory drugs, classical steroid anti-inflammatory agents, natural substances and drugs used for quite different goals. However, more compelling evidence supporting the involvement of neuroinflammation in PD pathogenesis relies on genome-wide association studies, which have found a genetic association with PD susceptibility in the human leukocyte antigen region (Nalls et al., 2014) and other polymorphisms related to the immune system including TNF, TNFR1, IL-1β, triggering receptor expressed on myeloid cells 2, IL-1 receptor antagonist and CD14 (Hirsch and Hunot, 2009; Rayaprolu et al., 2013). These studies highlight the importance of neuroinflammation-based animal models of PD, including those induced by injecting pro-inflammatory compounds such as thrombin (Carreño-Müller et al., 2003), tissue plasminogen activator (Villarán et al., 2009) or LPS within the SN (Castaño et al., 1998). Here, we took advantage of the intranigral injection of LPS in rats. LPS is the active endotoxin of Gram-negative bacteria that initiates the immune response after bacterial infection (Kielian and Blecha, 1995; Holst et al., 1996). LPS is a Toll-like receptor 4 ligand, present in microglial cells but not in neurons, that plays a critical role in the activation and proliferation of microglia (Beutler et al., 2001). It has been shown that subtoxic doses of LPS exacerbate disease progression in an animal model of PD (Godoy et al., 2008), thus supporting the hypothesis that brain inflammation may play a significant role in PD progression.

Metformin (metformin hydrochloride) is an extended used oral antidiabetic drug that reduces hepatic glucose production and decreases insulin resistance and the levels of plasma insulin in fasting. These actions seem to be mainly mediated by the activation of the AMP kinase (AMPK). Interestingly, several authors have shown that metformin also has anti-inflammatory properties, both peripherally (Mamputu et al., 2003; Li et al., 2005; Isoda et al., 2006) and centrally (Liu et al., 2014; Zhu et al., 2015; Ismaiel et al., 2016). So far, only a few studies have evaluated the effects of metformin in animal models of neurodegenerative disease. With these precedents, we wondered if metformin could be a suitable adjuvant for the treatment of PD. Therefore, the aim of this study was to determine the effect of metformin on the glial and neuronal cells of the SN of rats using the LPS animal model mentioned above. *In vitro* cell culture of BV2 cells was also used to study the activation of microglia. Our results show that metformin reduces microglial activation, at both cellular and molecular levels, but fails to protect dopaminergic neurons from the damage induced by LPS.

## Materials and Methods

### Cell Culture

For all cell culture experiments, we used murine microglial BV2 cell line. These cells were cultured in DMEM (Invitrogen, Carlsbad, CA, United States) supplemented with 10% heat-inactivated fetal bovine serum (Sigma-Aldrich, St. Louis, MO, United States), streptomycin (100 mg/ml), and penicillin (100 IU/ml), under 100% humidity and 5% CO_2_. Experiments were performed in reduced 5% heat-inactivated fetal bovine serum media.

For testing the effect of metformin on the pro- and anti-inflammatory phenotypes of microglial cells, BV2 cells were treated with LPS (pro-inflammatory phenotype inducer, 1 μg/ml, Sigma-Aldrich, St. Louis, MO, United States) or IL-4 (anti-inflammatory phenotype inducer, 20 ng/ml, Sigma-Aldrich, St. Louis, MO, United States), with and without metformin (1 mM, Dianben^®^ 1000 mg, MERK) for 12 h. The effect on production of ROS was measured by treating cells with LPS and/or metformin for 24 h. The effect on inflammasome activation was tested by Western blot and ELISA, treating cells with LPS and/or metformin for 3 h and then with ATP (1 mM, Sigma-Aldrich, St. Louis, MO, United States) as the second signal for inflammasome activation (Martinon et al., 2002).

### Animals and Surgery

Male albino Wistar rats (200–270 g) were used for these studies. The rats were kept at constant room temperature of 22 ± 1°C and relative humidity (60%) with a 12-h light–dark cycle with free access to food and water. Rats were anesthetized with isoflurane and positioned in a stereotaxic apparatus (Kopf Instruments, Tujunga, CA, United States) to conform to the brain atlas of Paxinos and Watson (1986). Injections into the SN were made 5.5 mm posterior, 1.8 mm lateral and 8.3 mm ventral to the bregma at day 1.

Experiments were carried out in accordance with the Guidelines of the European Union Directive (2010/63/EU) and Spanish regulations (BOE 34/11370-421, 2013) for the use of laboratory animals; the study was approved by the Scientific Committee of the University of Seville.

**FIGURE 1:**
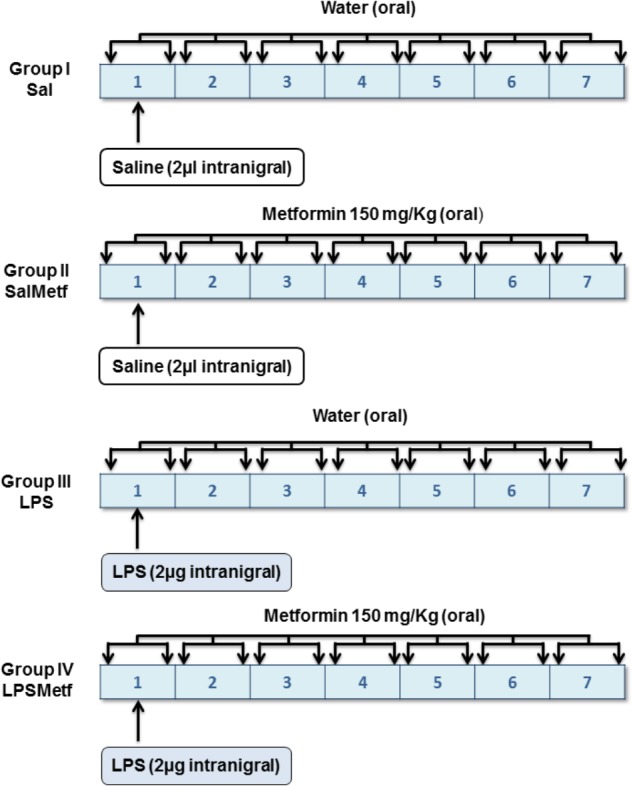
Experimental design: groups and treatments *in vivo*. Tap water or metformin were administered orally, twice a day for 7 days to the corresponding groups. Saline solution or LPS were injected intranigrally to the corresponding groups the 1st day of the experiment, before tap water or metformin were administered.

Animals were divided into four groups according to two different variables (Figure 1): solution administered orally by a plastic enteral feeding tube (tap water or 150 mg/kg of metformin dissolved in tap water, 4 mL/kg) and product injected in the SN (2 μL of saline solution or 2 μg of LPS dissolved in 2 μL of saline solution). In group I (the control group), animals received a single intranigral injection of saline solution (Monastral Blue inert tracer, 1% in saline solution; Sigma-Aldrich, St. Louis, MO, United States) the 1st day. Twice a day, animals received 4 mL/kg of tap water through the plastic tube for 7 days. Group II received a single intranigral injection of saline solution and two daily doses of metformin dissolved in tap water for 7 days. Group III received a single intranigral injection of LPS (from *Escherichia coli*, serotype 026:B6; Sigma-Aldrich, St. Louis, MO, United States) the 1st day and 4 mL/kg of tap water through the plastic tube twice a day for 7 days. Group IV received a single intranigral injection of LPS the 1st day and two daily doses of metformin dissolved in tap water for 7 days. The first dose of metformin or water was administrated 1 h before intranigral injection. All animals were sacrificed by perfusion or decapitation 7 days after the initiation of treatment, unless otherwise stated.

### Immunohistological Evaluation: Tyrosine Hydroxylase (TH), Glial Fibrillary Acidic Protein (GFAP) and OX-6

Animals used for immunohistochemistry completed 7 days of treatment with water/metformin. One week after the surgical procedure in the SN, animals were perfused through the heart under deep anesthesia (isoflurane) with 150–200 mL of 4% paraformaldehyde in phosphate buffer, pH 7.4. The brains were removed and then cryoprotected serially in sucrose dissolved in PBS, pH 7.4, first (24 h) in 10% sucrose, next (24 h) in 20% sucrose, and finally in 30% sucrose until they sank (2–5 days). Brains were then frozen in isopentane at -20°C, and sections of 20 μm thickness were cut on a cryostat at -20°C, and mounted in gelatine-coated slides. Primary antibodies used were rabbit-derived anti-TH (Sigma-Aldrich, St. Louis, MO, United States, 1:300), mouse-derived anti-GFAP (Chemicon International Inc.; 1: 300) and mouse-derived OX-6 (Serotec, Oxford, United Kingdom; 1: 200).

Sections were washed and then treated with 0.3% hydrogen peroxide in methanol for 15 min, washed again, and incubated in a solution containing TBS and 1% horse (for GFAP and OX-6) or goat (for TH) serum for 60 min in a humid chamber. Slides were drained and further incubated with the primary antibody in TBS containing 1% horse/goat serum and 0.25% Triton-X-100 for 24 h. Sections were then incubated for 2 h with biotinylated horse anti-mouse (for GFAP and OX-6) or biotinylated goat anti-rabbit (for TH) IgG (Vector, 1:200). The secondary antibody was diluted in TBS containing 0.25% Triton-X-100, and its addition was preceded by three 10-min rinses in TBS. Sections were then incubated with ExtrAvidin^®^-Peroxidase solution (Sigma-Aldrich, St. Louis, MO, United States, 1:100), for 1 h. The peroxidase was visualized with a standard diaminobenzidine/hydrogen reaction for 5 min.

### Immunohistochemistry Data Analysis

Analysis were made in a bounded region of the SN with a length of 300 microns in the anterior–posterior axis centered at the point of injection (5.5 mm with respect to bregma), that is, between 5.35 and 5.65 mm with respect to bregma. In each case, five sections per animal were used, with random starting point and systematically distributed through the anterior–posterior axis of the analyzed region. For the measurement of areas lacking GFAP immunoreactivity, the AnalySIS imaging software (Soft Imaging System GmbH, Münster, Germany) coupled to a Polaroid DMC camera (Polaroid, Cambridge, MA, United States) attached to a Leica light microscope (Leica Mikroskopie, Wetzlar, Germany) was used. For counting cells showing OX-6 immunoreactivity, a systematic sampling of the area occupied by the OX-6-positive cells in each section was made from a random starting point with a grid adjusted to count five fields per section. An unbiased counting frame of known area (40 μm × 25 μm = 1000 μm^2^) was superimposed on the tissue section image under a 100× oil immersion objective. The different types of OX-6-positive cells (displaying different shapes depending on their activation state) were counted as a whole and expressed as cells per mm^2^. The number of TH-positive neurons in the SN was estimated using a fractionator sampling design (Gundersen et al., 1988). Counts were made at regular predetermined intervals (*x* = 150 μm and *y* = 200 μm) within each section. An unbiased counting frame of known area (40 μm × 25 μm = 1000 μm^2^) was superimposed on the tissue section image under a 100× oil immersion objective. Therefore, the area sampling fraction is 1000/(150 × 200) = 0.033. The entire z-dimension of each section was sampled; hence, the section thickness sampling fraction was 1. In all animals, 20-μm sections, each 100 μm apart, were analyzed; thus, the fraction of sections sampled was 20/100 = 0.20. The number of neurons in the analyzed region was estimated by multiplying the number of neurons counted within the sample regions by the reciprocals of the area sampling fraction and the fraction of section sampled.

### Immunofluorescence

Animals were perfused and sections were prepared as described above. Incubations and washes for all the antibodies were in PBS, pH 7.4. All work was done at room temperature. For double-labeling of Iba-1 with IκB kinase β (IKKβ) or arginase, sections were blocked with PBS containing 1% goat serum (Vector, for Iba-1) and rabbit serum (Invitrogen, for IKKβ or arginase) for 1 h. The slides were washed three times in PBS and then incubated overnight at 4°C with either rabbit-derived anti-Iba-1 (1:300; Wako), goat-derived anti-IKKβ (1:300; Santa Cruz Biotechnology) and goat-derived anti-arginase (1:50; Santa Cruz Biotechnology), diluted in PBS containing 1% goat/rabbit serum and 0.25% Triton X-100. Sections were incubated with goat anti-rabbit secondary antibody conjugated to Alexa Fluor^®^ 594 (1:200, for Iba-1; Invitrogen) and rabbit anti-goat secondary antibody conjugated to fluorescein (1:200, for IKKβ and arginase; Vector) for 1 h at 22 ± 1°C in the dark and its addition was preceded by three 10-min rinses in PBS. As a control, another set of experiments was performed where the sections were only incubated with the Iba-1 antibody and then visualized with both filters. No signal was detected when Iba-1 alone with fluorescein filter was used (photomicrograph not shown). The same was true with IKKβ or arginase when Alexa Fluor^®^ 594 filter was used. Fluorescence images were acquired using a confocal laser scanning microscope (Zeiss LSM 7 DUO) and processed using the associated software package (ZEN 2010).

### Real-Time RT-PCR

BV2 cells were seeded at a concentration of 1 × 10^5^ cells/well in 12-well plates (Sarstedt AG & Co., Germany) and then treated with LPS (1 μg/ml) or IL-4 (20 ng/ml), with and without metformin (1 mM). Treatments were conducted for 12 h. At the end of the treatment, cells were collected in TRIsure^TM^ (Bioline, USA Inc.) and RNA was extracted from cells following the manufacturer’s protocol. cDNA was synthesized using 1 μg of extracted RNA using Revert Aid First Strand cDNA Synthesis Kit (Thermo Fisher Scientific) in 20 μL reaction volume as described by the manufacturer.

Animals used for RT-PCR followed the procedure described above but were sacrificed by decapitation 6 h after LPS/saline injection in the SN, thus they only received a single dose of metformin/water orally 1 h before the LPS/saline injection.

SN was dissected from each rat, snap frozen in liquid nitrogen and stored at -80°C. Total RNA was extracted from the rat SN of different treatments using RNeasy^®^ kit (Qiagen). cDNA was synthesized from 1 μg of total RNA using Revert Aid First Strand cDNA Synthesis Kit (Thermo Fisher Scientific) in 20 μL reaction volume as described by the manufacturer.

Real-time PCR was performed using 5 μL SensiFAST^TM^ SYBR NO-ROX KIT (Bioline, United States), 0.4 μL of each primer, and 4.2 μL cDNA to get to a final reaction volume of 10 μL for 384-well plate. Controls were carried out without cDNA. Amplification was run in a Lightcycler^®^ 480 Instrument II (Roche) thermal cycler at 95°C for 2 min followed by 40 cycles consisting of a denaturation phase for 5 s at 95°C, followed by a second phase of hybridization at 65°C for 10 s, and a final phase of elongation at 72°C for 20 s. The process was terminated by a final step of 7 min at 72°C. Analysis confirmed a single PCR product. β-actin served as reference gene and was used for samples normalization. The cycle at which each sample crossed a fluorescence threshold (*C*t value) was determined, and the triplicate values for each cDNA were averaged. The primer sequences for IL-1β, TNF-α, iNOS, arginase, IL-10, IL-6, CX3CR1, CD200, MCP-1, the heme-binding membrane-bound phagocytic (NAPDH) subunits (p22^phox^ and gp91^phox^) and β-actin are shown in Tables 1, 2.

**Table 1:** RT-PCR primers *in vitro*.

mRNA	Primers
IL-1β	F: 5′-TTGACGGACCCCAAAAGATG-3′
	R: 5′-AGAAGGTGCTCATGTCCTCA-3′
TNF-α	F: 5′-AGCCCACGTCGTAGCAAACCACCAA-3′
	R: 5′-AACACCCATTCCCTTCACAGAGCAAT-3′
iNOS	F: 5′-CTTTGCCACGGACGAGAC-3′
	R: 5′-TCATTGTACTCTGAGGGCTGAC-3′
Arginase	F: 5′- TCA CCT GAG CTT TGA TGT CG -3′
	R: 5′- CTG AAA GGA GCC CTG TCT TG -3′
IL-10	F: 5′- CCAAGCCTTATCGGAAATGA -3′
	R: 5′- TTTTCACAGGGGAGAAATCG -3′
β-actin	F: 5′-CCA CAC CCG CCA CCA GTT CG-3′
	R: 5′-CCC ATT CCC ACC ATC ACA CC-3′
p22^phox^	F: 5′-GAATTCCGATGGGCAGATCGA-3′
	R: 5′-GGAQTCCCGTCACACGACCTCA-3′
gp91^phox^	F:5′-GCACAGCCAGTAGAAGTAGATCTTT-3′
	R: 5′-GCTGGGATTGGAGTCACG-3′


**Table 2:** RT-PCR primers *in vivo.*

mRNA	Primers
TNF-α	F: 5′-TACTGAACTTCGGGGTGATTGGTCC-3′
	R: 5′-CAGCCTTGTCCCTTGAAGAGAACC-3′
IL-6	F: 5′-AAAATCTGCTCTGGTCTTCTGG-3′
	R: 5′-GGTTTGCCGAGTAGACCTCA-3′
IL-1β	F: 5′-CAGGATGAGGACATGAGCACC-3′
	R: 5′-CTCTGCAGACTCAAACTCCAC-3′
iNOS	F: 5′-CCTCCTCCACCCTACCAAGT-3′
	R: 5′-CACCCAAAGTGCTTCAGTCA-3′
CD200	F: 5′-TGTTCCGCTGATTGTTGGC-3′
	R: 5′- ATGGACACATTACGGTTGCC-3′
CX3CR1	F: 5′-GGC CTT GTC TGA TCT GCT GTT TG-3′
	R: 5′- AAT GCT GAT GAC GGT GAT GAA GAA-3′
MCP-1	F: 5′-AGCATCCACGTGCTGTCTC-3′
	R: 5′-GATCATCTTGCCAGTGAATGAG-3′
β-actin	F: 5′-TGTGATGGTGGGAATGGGTCAG-3′
	R: 5′-TTTGATGTCACGCACGATTTCC-3′


### Western Blot

In order to obtain proteins from BV2 cell line, cells were seeded in 6-well plates (Sarstedt AG & Co., Germany) at a seeding concentration of 200 × 10^3^ cell/well and left for 24 h at 37°C to reach the required confluency. Then, cells were treated with LPS (1 μg/ml) and/or metformin (1 mM) for 3 h, followed by ATP (1 mM) for 1 h. At the end of the treatment which lasted for 4 h cells were collected in 50 μl RIPA buffer (Sigma-Aldrich, St. Louis, MO, United States) with the aid of a scraper after being washed three times with ice-cold PBS. Cells lysate was sonicated (10% amplitude, 10 s, on ice) using a probe sonicater (Hielscher Ultrasound Technology). All steps were carried out on ice. Protein concentrations were determined using Pierce^TM^ BCA protein assay kit (Thermo Fischer Scientific).

Animals used for Western blot followed the procedure described above and were sacrificed by decapitation 7 days after LPS/saline injection in the SN. Proteins were extracted from the SN of rat brains through the addition of 350 μl RIPA buffer along with 3.5 μl protease inhibitor followed by sonication (10% amplitude, 10 s, on ice). Afterwards, samples were heated in the thermoblock for 5 min at 37°C, left 20 min on ice and centrifuged (4°C, 13,000 × *g*, 15 min). The clear supernatant which contained the proteins was collected and quantified with Pierce^TM^ BCA protein assay kit (Thermo Fischer Scientific).

Proteins (40 μg) were separated on 10% SDS-PAGE gel, subjected to electrophoresis at 150 V, and then transferred using Trans-Blot^®^ Turbo^TM^ Transfer System (Bio-Rad Laboratories, Hercules, CA, United States) onto 0.45 μm nitrocellulose membrane (Bio-Rad Laboratories, Hercules, CA, United States) for 7 min.

After blocking with 5% skimmed milk diluted in TBST (0.05% tween 20 in TBS) for 1 h, membranes were incubated with a primary antibody against phosphorylated and non-phosphorylated forms of the MAPKs family proteins p38 and JNK (Santa Cruz Biotechnology) for *in vivo* experiment, and against IL-1β (R&D Systems, Minneapolis, United States) for *in vitro* experiment. Antibodies of non-phosphorylated forms of MAPKs and the housekeeping GAPDH were used at a dilution of 1:1000 in 5% skimmed milk dissolved in TBST, while the antibodies of the phosphorylated forms of MAPKs and IL-1β were used at a dilution of 1:500 in 5% skimmed milk dissolved in TBST. Membranes were incubated with these antibodies for 24 h at 4°C. Membranes were washed 4 times (10 min each) with TBST, then incubated for 1 h with the secondary antibody (1:5000) in 5% skimmed milk dissolved in TBST against the specific primary antibody at room temperature. Another 4 washes with TBST (10 min each) were done before visualizing the bands with Clarity^TM^ and Clarity Max^TM^ western ECL Blotting Substrates (Bio-Rad Laboratories, Hercules, CA, United States) using Amersham Imager 600 Imager (General Electric, United States). These images were later analyzed using the ImageJ software.

### Production of Reactive Oxygen Species

To determine the level of oxidative stress produced in viable BV2 cell cultures 35 × 10^3^ cells/well were seeded in Ibidi^®^ plates (Invitrogen, Carlsbad, CA, United States) and incubated for 24 h at 37°C. Then cells were treated with metformin (1 mM) and/or LPS (1 μg/ml) for 24 h. At the end of the treatment a membrane permeable reagent was added (CellROX^®^ Deep Red Reagent) at each well. This reagent is a fluorogenic probe that exhibits fluorescence signal in the presence of ROS in the cell cytoplasm.

The test was performed following the manufacturer instructions. The reactive was added to the treated cells at a final concentration of 5 mM, and incubated at 37°C for 30 min. Subsequently, the media was removed and cells were washed with PBS three times. Confocal microscope (Zeiss LSM 7 DUO) was employed to acquire images of the treated cells. Images of five fields per condition were captured. These images were later analyzed using the ImageJ software. In each image, the fluorescence per cell corresponding to ROS production was calculated using the following formula:

ROS = (*I*_T_ - (*A*_T_^∗^*I*_B_)/*A*_B_))/*N*, where *I*_T_ is total intensity of the image, *A*_T_ is total area of the image, *I*_B_ is background intensity, *A*_B_ is background area and *N* is number of cells.

### Enzyme-Linked Immunosorbent Assay

BV2 cells were seeded in 24-well plates (Sarstedt AG & Co., Germany) at a concentration of 75 × 10^3^ cells per well for 24 h. Then, cells were treated with metformin (1 mM) and/or LPS (1 μg/mL) and incubated at 37°C for 3 h. After this time ATP (1mM) was added at each well for 1 h. At the end of the treatment, the supernatant was collected, centrifuged at 400 × *g* 5 min at 4°C to remove cell debris and stored at -80°C for subsequent use. 100 μl of the supernatant was used for the measurement of IL-1β production by ELISA following the manufacturer’s instructions (Invitrogen, Mouse IL1β ELISA Ready-SET-GO! Thermo Fisher Scientific). All standards and samples were run in duplicates. The supernatant was spun before removing cell debris. Samples were diluted in a proportion of 1:1 in ELISA/ELISASPOT Diluent (1×), except samples from cells treated with LPS alone which were diluted in a proportion of 1:2.

### Statistical Analysis

Results are expressed as mean ± SD. Means were compared by One-Way ANOVA followed by the LSD test for *post hoc* multiple range comparisons. An alpha level of 0.05 was used. The Statgraphics Plus 3.0 statistical package was used for the analyses.

## Results

### Metformin Treatment Reduced Microglial Activation *in vitro*

**FIGURE 2:**
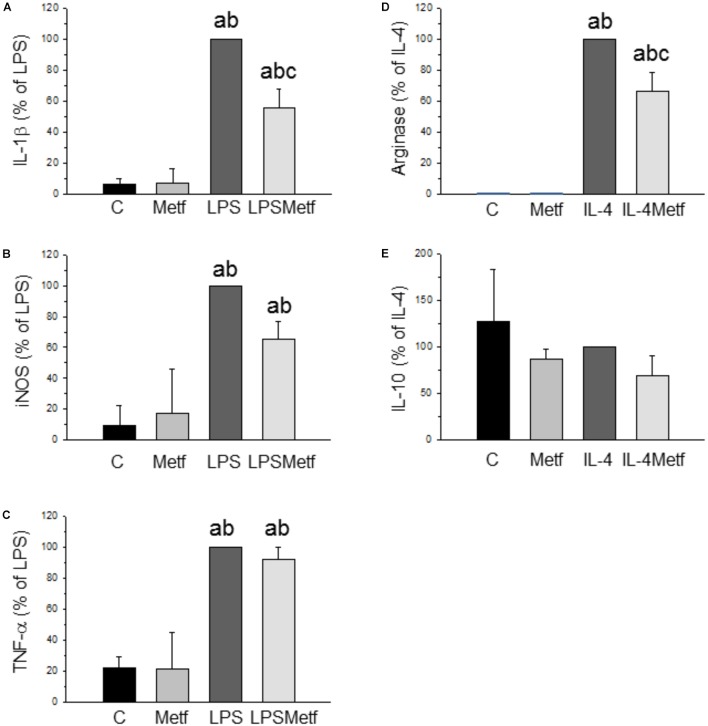
Expression levels of the mRNAs of IL-1β **(A)**, iNOS **(B)**, TNF-α **(C)**, arginase **(D)**, and IL-10 **(E)** in BV2 cells. Total RNA was extracted from cells after 12 h of treatment (LPS for IL-1β, iNOS and TNF-α; IL-4 for arginase and IL-10). Results are mean ± SD of three independent experiments, normalized to β-actin and expressed as percentage relative to the LPS or IL-4 group. Statistical significance (one-way ANOVA followed by the LSD *post hoc* test for multiple comparisons): a, compared with the control group; b, compared with the metformin group; c, compared with the LPS (or IL-4; **D,E**) groups; *p* < 0.001. (C, control cells without treatment; Metf, cells treated with metformin; LPS, cells treated with LPS; IL4, cells treated with IL-4; LPSMetf, cells treated with LPS and metformin; IL4Metf, cells treated with IL-4 and metformin).

BV2 cells were treated with either LPS to induce a pro-inflammatory phenotype or IL-4 to induce an anti-inflammatory phenotype. After treatment with LPS, a strong induction of the three pro-inflammatory mediators studied was found, ranging from 348% for TNF-α to 1438% for IL-1β (with respect to control levels; *p* < 0.001; Figures 2A–C). Metformin treatment largely prevented the LPS-induced mRNA expression levels of IL-1β (56 ± 11.5% vs. LPS group; *p* < 0.001; Figure 2A). In contrast, metformin failed to alter the LPS-induced mRNA levels of nitric oxide synthase (iNOS) and TNF-α (Figures 2B,C). When BV2 cells were treated with IL-4, a strong induction of arginase (20000-fold compared to the control group, *p* < 0.001; Figure 2D) was found. This IL-4-induced upregulation was largely prevented by metformin treatment (63.3 ± 12.5% vs. IL-4 group; *p* < 0.001; Figure 2D). IL-10 mRNA levels were unaffected in response to IL-4 (Figure 2). No effect was found in the amounts of IL-10 mRNA when metformin was added to IL-4-treated cells (Figure 2E).

### Metformin Treatment Decreased the Production of Reactive Oxygen Species

**FIGURE 3:**
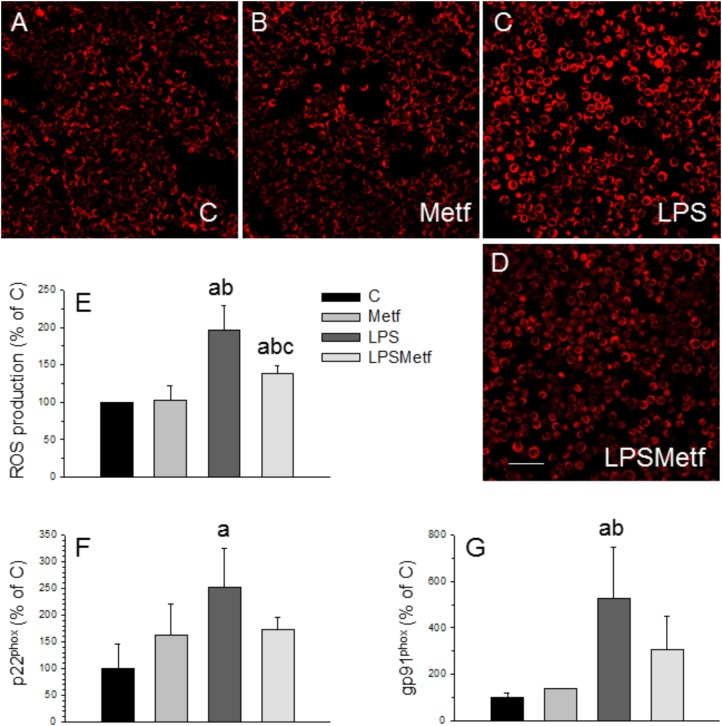
Effect of LPS and metformin on ROS production and expression levels of NADPH oxidase subunits in BV2 cells. ROS were measured 24 h after the different treatments assayed: **(A)** control; **(B)** metformin; **(C)** LPS; **(D)** LPS plus metformin. **(E)** Quantification of ROS production. Results are mean ± SD of 3 independent experiments, expressed as percentage of control. Scale bar, 50 μm. Expression levels of p22^phox^ **(F)** and gp91^phox^ **(G)** mRNAs were measured by RT-PCR in BV2 cells from the different treatments assayed (C, control cells without treatment; Metf, cells treated with metformin; LPS, cells treated with LPS; LPSMetf, cells treated with LPS and metformin). Results are mean ± SD of three independent experiments for the Metf, LPS, and LPSMetf groups, and 4 (gp91^phox^) and 5 (p22^phox^) independent experiments for the C group. Results are normalized to β-actin and expressed as percentage relative to the control group. Statistical significance (one-way ANOVA followed by the LSD *post hoc* test for multiple comparisons): a, compared with the control group; b, compared with the Metf group; c, compared with the LPS group. *p* < 0.001 for **(E)** and *p* < 0.05 for **(F,G)**.

Taking into account that the main source of ROS in microglial cells is the NADPH oxidase, we next sought to measure the level of oxidative stress after the treatments with LPS and metformin. We took advantage of a fluorogenic probe that exhibits fluorescence signal in the presence of ROS. As expected, LPS induced a two-fold increase of ROS production (197.1 ± 31.6% with respect to the control, *p* < 0.001; Figures 3A–C,E), whereas metformin decreased it around 30% (138.8 ± 9.7% with respect to the control, *p* < 0.001; Figures 3D,E).

### Metformin Treatment Prevented the Induction of NADPH Oxidase by LPS

In microglial cells, ROS are mainly derived from NADPH oxidase (Block, 2008; Gao et al., 2012). This enzyme complex consists on several subunits, including cytosolic subunits (p40^phox^, p47^phox^, and p67^phox^), the membrane bound cytochrome b558 (p22^phox^), the heme binding enzymatic subunit (gp91^phox^) and the Rac G-protein (Dohi et al., 2010). After a pathogenic stimulus, the different subunits of the NADPH oxidase associates leading to its activation (Block, 2008; Gao et al., 2012). Therefore, the expression levels of the p22^phox^ and gp91^phox^ subunits of the NADPH oxidase enzyme were measured by RT-PCR to study the effect of metformin on the oxidative stress induced by LPS in BV2 cells. mRNA levels of p22^phox^ and gp91^phox^ subunits significantly increased after LPS treatment (251.1 ± 74.2% and 524.8 ± 222.7% control levels, respectively; Figures 3F,G; *p* < 0.05). Metformin treatment prevented the LPS-induced mRNA levels of both p22^phox^ and gp91^phox^ subunits, which remained not different to control values (173.2 ± 21.7% and 308.7 ± 136.9%, *p* < 0.05, Figures 3F,G).

### Metformin Treatment Decreased the Number of Activated Microglial Cells *in vivo*

**FIGURE 4:**
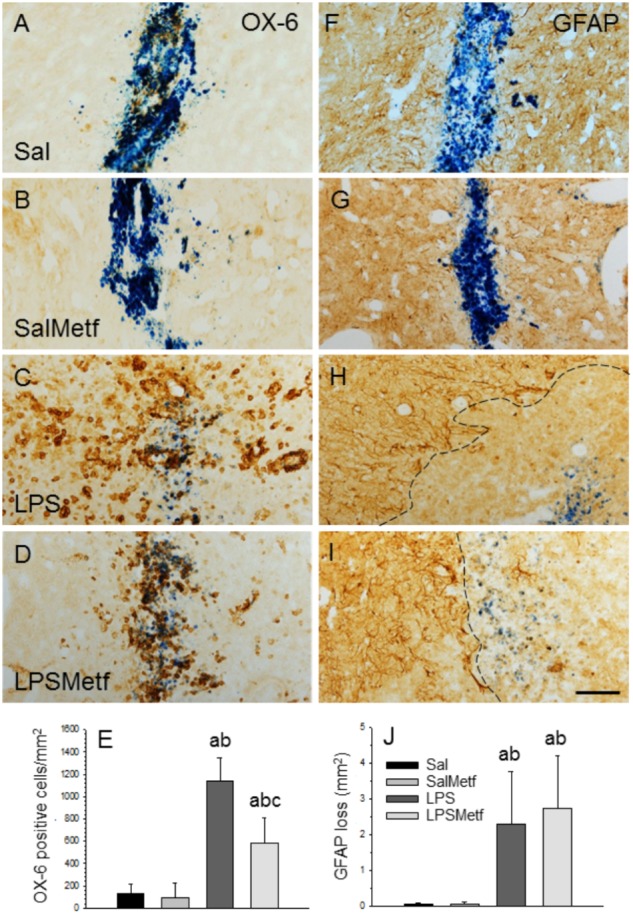
Effect of metformin and LPS on microglia and astroglia, evaluated by immunohistochemistry with the OX-6 (microglia; **A–D**) and the anti-GFAP (astroglia, **F–I**) antibodies. **(A,F)** Animals injected with saline solution in the SN plus oral administration of water; **(B,G)** animals injected with saline solution in the SN plus oral administration of metformin; **(C,H)** animals injected with LPS in the SN plus oral administration of water; **(D,I)**, animals injected with LPS in the SN plus oral administration of metformin. The activation of microglia induced by LPS **(C)** was partially prevented by metformin **(D)**. GFAP immunohistochemistry showed an important loss of astroglia induced by LPS **(H)** that was not prevented by metformin **(I)**. The dashed line in **(H,I)** marks the left boundary of the area lacking GFAP staining. Metformin alone had no effect on either microglia **(B)** or astroglia **(G)**. **(E)** Quantification of the density of OX-6 positive cells. Results are mean ± SD of four independent experiments, expressed as number of cells per mm^2^. **(J)** Quantification of the areas lacking GFAP immunostaining. Results are mean ± SD of four independent experiments, expressed as mm^2^. Statistical significance (one-way ANOVA followed by the LSD *post hoc* test for multiple comparisons): a, compared with the control group; b, compared with the SalMetf group; c, compared with the LPS group; *p* < 0.01 for **(E)** and *p* < 0.05 for **(J)**. Scale bar, 50 μm.

Once we demonstrated that metformin treatment reduces microglial activation in response to LPS *in vitro*, we next studied whether this was also the case in the SN after LPS injection *in vivo* at morphological and molecular levels. Immunohistochemistry was performed with a marker of reactive microglia (OX-6) to evaluate the effect of metformin on the LPS-associated neuroinflammation. Both control and metformin groups showed a similar mild OX-6 immunoreactivity around the injection tract (Figures 4A,B,E). In contrast, the LPS-treated group showed a clear microglial activation, nearly 10 times higher than the control group (1141.9 ± 207.8 cells/mm^2^, *p* < 0.01; Figures 4C,E). Metformin hindered LPS-induced microglia activation by almost half (584.2 ± 230.1 cells/mm^2^, *p* < 0.01; Figures 4D,E). Therefore, metformin significantly decreased the inflammation caused by LPS.

### Metformin Treatment Failed to Protect Against LPS-Induced Damage to Astrocytes

In previous works, we have shown that the intranigral injection of LPS induces a significant loss of GFAP-reactive astrocytes extending from the injection site (Castaño et al., 1998; Castańo et al., 2002; Herrera et al., 2000). Consequently, we next analyzed the astroglial population in our experimental conditions. As expected, in absence of LPS, a slight astrogliosis around the injection site was found in saline-injected animals from both control and metformin-treated animals without loss of GFAP immunostaining (Figures 4F,G,J). In confirmation of previous reports, a significant loss of GFAP-reactive astrocytes extending from the injection site was evident in LPS-injected animals (2.30 ± 1.48 mm^2^; *p* < 0.05 vs. controls). The area exhibiting GFAP loss was surrounded by highly reactive astrocytes (Figures 4H,J). Metformin treatment failed to prevent the loss of astrocytes in response to LPS injection (2.78 ± 1.48 mm^2^; Figures 4I,J).

### Metformin Treatment Attenuated the Expression Levels of Several Inflammatory Mediators After LPS Injection *in vivo*

**FIGURE 5:**
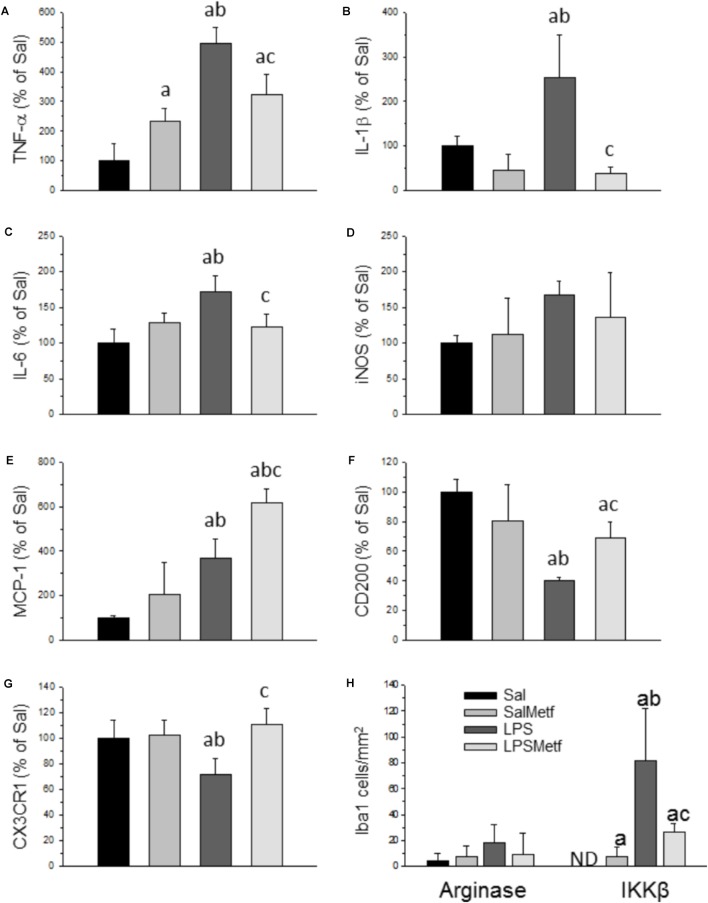
Effect of metformin and LPS on the expression of TNF-α **(A)**, IL-1β **(B)**, IL-6 **(C)**, iNOS **(D)**, MCP-1 **(E)**, CD200 **(F)**, and CX3CR1 **(G)** mRNAs in the SN of rats from the different treatments assayed, measured by RT-PCR. Sal, animals injected with saline solution in the SN plus oral administration of water; SalMetf, animals injected with saline solution in the SN plus oral administration of metformin; LPS, animals injected with LPS in the SN plus oral administration of water; LPSMetf, animals injected with LPS in the SN plus oral administration of metformin. Results are mean ± SD of 4 independent experiments, normalized to β-actin and expressed as percentage relative to the control (Sal) group. **(H)** Quantification of the effect of metformin and LPS on Iba-1, IKKβ and arginase immunostaining in the SN. Arginase is poorly induced in Iba-1-labeled microglial cells after the treatments assayed. The expression of IKKβ is induced by LPS in Iba-1 positive cells, an effect decreased by metformin. Results are mean ± SD of three independent experiments expressed as cells per mm^2^. Statistical significance (one-way ANOVA followed by the LSD *post hoc* test for multiple comparisons): a, compared with the Sal group; b, compared with the SalMetf group; c, compared with the LPS group; *p* < 0.001.

Activated microglia expresses pro-inflammatory cytokines and several pro-inflammatory mediators. Analysis by real-time PCR showed that the mRNAs for the pro-inflammatory cytokines TNF-α, IL-1β, and IL-6 were induced in the SN 6 h after LPS injection (496.3 ± 53.0%, 243,3 ± 96%, and 172.5 ± 21.9% control levels, respectively; *p* < 0.01; Figures 5A–C). Moreover, LPS treatment induced the expression of MCP-1 (369.0 ± 87.0% with respect to the control; *p* < 0.001; Figure 5E).

CD200 and CX3CR1 have shown to have inhibitory actions on brain microglia. Hence, we decided to study these molecules in our experimental conditions to seek further explanations of how metformin induces an attenuated effect after LPS treatment. Our PCR analysis showed that the expression of CD200 and CX3CR1 mRNAs is decreased with respect to the control group (40.0 ± 2.5% and 71.1 ± 12.7%, respectively; *p* < 0.001 for CD200 and *p* < 0.05 for CX3CR1; Figures 5F,G).

Metformin significantly partially prevented the LPS-induced increases in the mRNA levels of inflammatory markers studied including TNF-α (322.9 ± 67.6% controls); IL-1β (38.1 ± 13.5% controls) and IL-6 (122.5 ± 18.8% controls) (Figures 5A–C; *p* < 0.01). On the contrary, metformin treatment further increased the LPS-induced MCP-1 levels (619.7 ± 59.2% controls, *p* < 0.001) and, interestingly, prevented the LPS-induced decreased mRNA levels of CD200 (68.0 ± 10.5%, *p* < 0.001) and CX3CR1 (110.2 ± 12.1% controls, *p* < 0.05) (Figures 5E–G).

### Metformin Attenuated the LPS-Induced Microglial Pro-inflammatory Phenotype

To study the effect of metformin on the phenotype of microglial cells challenged by LPS, a double immunohistochemistry using a pan-microglia marker (Iba-1) with either a pro-inflammatory (IKKβ) or an anti-inflammatory (arginase) marker was performed (Figure 5H and Supplementary Figures S1, S2). After treatment with LPS, most microglial cells co-localized with IKKβ (Figure 5H; *p* < 0.001) but not with arginase (scarce co-localization), thus supporting the appearance of a strong microglial pro-inflammatory phenotype. When animals were treated orally with metformin, most microglial cells expressed IKKβ, whereas no co-localization appeared with arginase. Therefore, LPS also induces a pro-inflammatory phenotype even in metformin-treated animals.

### Metformin Treatment Decreased the Activation of the Inflammasome

**FIGURE 6:**
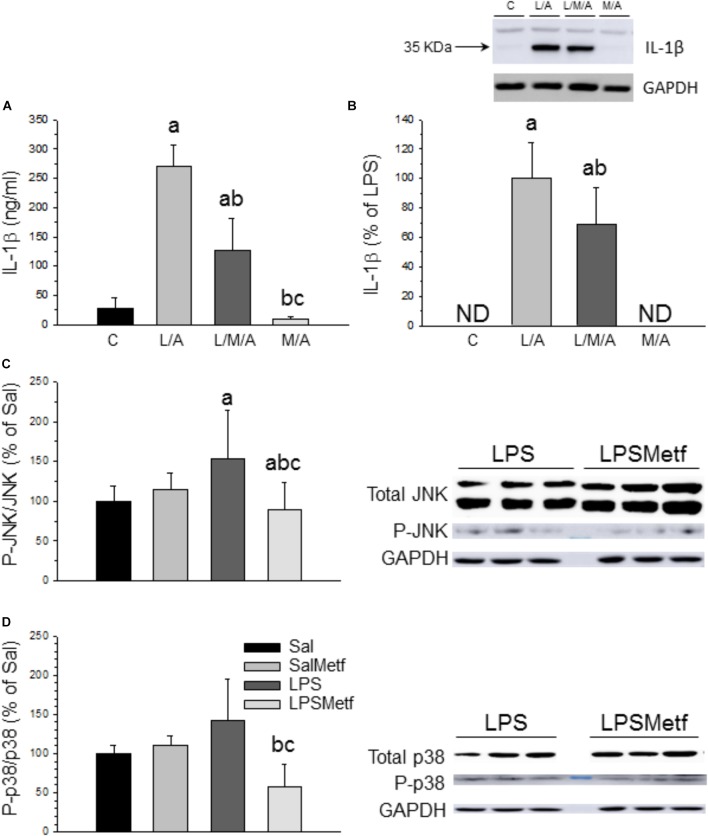
Effect of metformin and LPS on inflammasome activation in BV2 cells and expression of JNK, P-JNK, p38, and P-p38 proteins in the SN. **(A)** Extracellular IL-1β measured by ELISA. **(B)** Intracellular IL-1β measured by Western blot. Results are mean ± SD of three independent experiments for all groups, expressed as ng/ml **(A)**, and three independent experiments for all groups, expressed as percentage of the L/A group **(B)**; intracellular amounts are normalized to GAPDH expression. Statistical significance (one-way ANOVA followed by the LSD *post hoc* test for multiple comparisons): a, compared with the control group; b, compared with the L/A group; c, compared with the L/M/A group; *p* < 0.001. **(C)** Control group; L/A, cells treated with LPS and ATP; L/M/A, cells treated with a combination of LPS, metformin and ATP; M/A, cells treated with a combination of metformin and ATP; ND, not detected. Proteins from the SN of rats under the different treatments assayed were separated by electrophoresis and transferred to nitrocellulose membranes, and stained using anti-JNK, anti-P-JNK, anti-p38 and anti-P-p38 antibodies. Total optical density of each band was calculated. Results are mean ± SD of seven independent experiments for groups Sal, LPS and LPSMetf and eight independent experiments for group SalMetf **(C)**, and three independent experiments for groups Sal and LPS and four independent experiments for groups SalMetf and LPSMetf **(D)**, expressed as P-JNK/JNK **(C)** and P-p38/p38 **(D)** intensity ratios (normalized to GAPDH expression) relative to the control (Sal) group. Statistical significance (one-way ANOVA followed by the LSD *post hoc* test for multiple comparisons): a, compared with the control (Sal) group; b, compared with the SalMetf group; c, compared with the LPS group; *p* < 0.01.

In microglial cells the nucleotide-binding oligomerization domain-like receptor protein containing a pyrin domain 3 (NLRP3) inflammasome activation follows a two-signal model; a first signal (priming response) is, for instance, provided by Toll-like receptor (TLR) activation leading to NLRP3 and pro-IL-1β expression; a second signal (activation) is triggered, among others, by ATP or nigericin leading to inflammasome assembly and caspase-1 activation (He et al., 2016). Consequently, LPS followed by ATP treatment is a well-established strategy to activate the NLRP3 inflammasome (Martinon et al., 2002; Li et al., 2018). In our experimental conditions, we used LPS as first signal and ATP as the second one. We also have included another set of experiments only with LPS as a first signal. When activated, NLRP3 inflammasome induces the maturation of pro IL-1β into IL-1β through a caspase 1 dependent mechanism, leading to the release of this cytokine to the extracellular medium. We wanted to know if metformin treatment was able to decrease inflammasome activation after LPS/ATP treatment in BV2 cells. ELISA and Western blot analysis confirmed that LPS/ATP treatment increased the expression of both extracellular and intracellular IL-1β, whereas metformin significantly prevented this effect (around 50% vs. LPS/ATP group; *p* < 0.001; Figures 6A,B and Supplementary Figures S3, S4). Similar results were found when BV2 were challenged with LPS alone (in absence of ATP treatment) (Supplementary Figures S3, S4), thus supporting that TLR4 stimulation alone may drive inflammasome priming and activation in BV2 cells without the need for a secondary signal. This distinctive one-step pathway of inflammasome activation is typically seen in monocytes and have been recently seen in recent studies using BV2 cells (Carta et al., 2011; Huang M.Y. et al., 2018; Yang et al., 2018).

### Metformin Effects on Microglial Activation Are Mediated by MAPK

As a further step, activation of MAPKs was determined by Western blot analysis using specific antibodies against the phosphorylated and non-phosphorylated forms of JNK and p38. As expected, LPS treatment increased the ratio P-JNK/JNK (153.7 ± 60.5%; *p* < 0.05 compared with control animals; Figure 6C and Supplementary Figures S5, S6), whereas metformin treatment abolished this effect (89.0 ± 33.7% of control values; *p* < 0.05 compared with the LPS group). LPS increased the ratio P-p38/p38 (141.52 ± 53.8%; *p* < 0.05 compared with control animals), whereas metformin treatment reduced it (57.9 ± 29.2% of control values; *p* < 0.05 compared with the LPS group; Figure 6D and Supplementary Figure S5).

### Effect of Metformin and LPS on the Dopaminergic System

**FIGURE 7:**
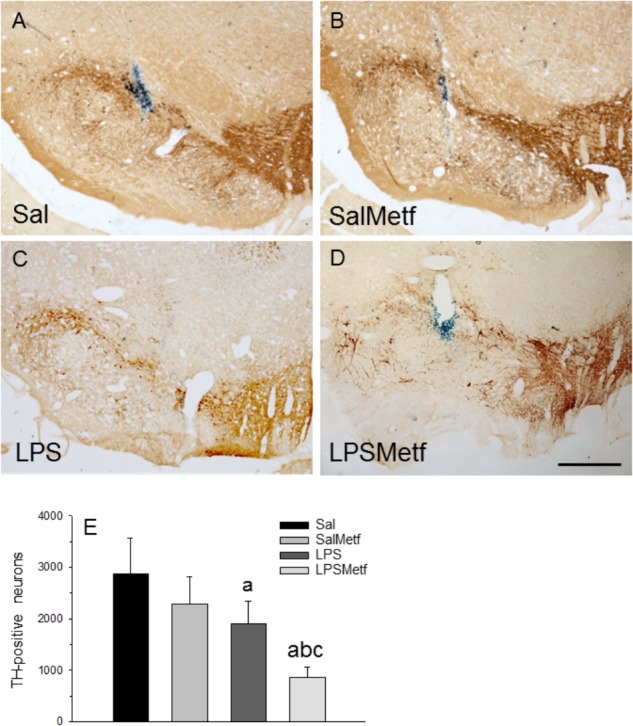
Effect of metformin and LPS on dopaminergic neurons. TH immunoreactivity was not affected by the injection of saline solution in the SN of rats **(A)** and the administration of metformin **(B)**, but decreased after the injection of 2 μg of LPS **(C)**. Loss of TH immunoreactivity induced by LPS increased in the animals treated orally with metformin **(D)**. Scale bar: 500 μm. **(E)** Quantification of the number of TH-positive cells. Results are mean ± SD of four independent experiments, expressed as TH-positive cells within the bounded area of the SN. Statistical significance (one-way ANOVA followed by the LSD *post hoc* test for multiple comparisons): a, compared with the control (Sal) group; b, compared with the SalMetf group; c, compared with the LPS group; *p* < 0.01.

Finally, we have evaluated the integrity of the nigro-striatal dopaminergic neurons in terms of immunoreactivity of TH, the rate-limiting enzyme in the synthesis of dopamine. Oral administration of metformin (Figures 7B,E) had no effect on the number of dopaminergic neurons with respect to the control group (2882 ± 685 and 2294 ± 530 for control and metformin, respectively; Figures 7A,B,E). Intranigral administration of LPS reduced the number of neurons to 66.1% of control values (Figures 7C,E; *p* < 0.01). Interestingly, metformin not only failed to protect against the LPS-induced death of dopaminergic neurons, but exacerbated the damage (29.6% of control value; Figures 7D,E).

## Discussion

In this study, we have evaluated the effect of metformin on microglia polarization, inflammasome activation, free radical production and main immune checkpoints, including CX3CL1/CX3CR1R and CD200/CD200R in response to LPS, *in vivo* and *in vitro*. Our study demonstrates that metformin exhibits potent MAPK-dependent anti-inflammatory effects on microglia in response to TLR activation. However, metformin treatment failed to counteract the death of dopaminergic neurons induced by a single intranigral injection of LPS.

It is becoming evident that microglia may display a varied range of reaction states including pro-inflammatory, anti-inflammatory and the recent disease-associated phenotype (reviews, Keren-Shaul et al., 2017; Krasemann et al., 2017). As a first step, we analyzed the effect of metformin on two well defined methods of microglia polarization based on treatment of BV2 cells with either LPS (pro-inflammatory stimulus) or IL-4 (anti-inflammatory stimulus). Analysis of specific microglia markers typically associated to these phenotypes (i.e., IL-1β or arginase) demonstrated an overall immunosuppressive role of metformin in driving the switch of homeostatic microglia to both LPS-induced pro-inflammatory and IL-4-induced anti-inflammatory phenotypes. Our results not only confirm the anti-inflammatory properties of metformin (Isoda et al., 2006; Łabuzek et al., 2010; Gu et al., 2014) but also reveals an overall immunosuppressive role on microglial cells.

We next studied the effect of metformin on different pro-inflammatory microglia-related pathways associated to neurodegeneration, including the NADPH oxidase system (Belarbi et al., 2017) and the NLRP3 inflammasome (Heneka et al., 2018). NADPH activation generates superoxide, which is released and then either transformed into H_2_O_2_ by extracellular superoxide dismutase or reacts with NO to produce peroxynitrite (Hickman et al., 2018). We followed a double *in vitro* approach in BV2 cells challenged with LPS: (i) measurement of ROS and (ii) measurement of levels of the p22^phox^ and gp91^phox^ subunits of the NADPH oxidase. In keeping with the overall immunosuppressant action of metformin, it highly prevented both, LPS-induced formation of ROS and up-regulation of p22^phox^ and gp91^phox^ subunits of the NADPH oxidase complex.

Inflammasomes are molecular platforms mediating inflammatory responses through the maturation and release of IL-1β through a caspase-1-dependent mechanism (Zhou et al., 2010). NLRP3 is one of the most intensively investigated inflammasomes, controlling disease progression and inflammatory responses (Yang et al., 2014). It has been shown that metformin is able to decrease the activation of NLRP3 in various tissues (Lee et al., 2013; Bullón et al., 2016; Li et al., 2016). Different studies have reported rather contradictory effects of metformin on NLRP3 inflammasome activation in macrophages, including inhibition (Kelly et al., 2015) and activation (Zha et al., 2016). To our knowledge, the effect of metformin on NLRP3 activation in microglial cells has never been studied. We provide evidence that metformin inhibits the NLRP3 inflammasome priming and activation in microglia. Overall, our data demonstrate a robust anti-inflammatory effect of metformin on LPS-stimulated microglial cells.

Given the strong effect of metformin on immune-related neurodegeneration pathways, we analyzed the potential anti-inflammatory effect of metformin in an *in vivo* model of PD associated to neuroinflammation. To date only a few studies have analyzed the effect of metformin on *in vivo* PD animal models, which were restricted to that based on MPTP injection (Patil et al., 2014; Bayliss et al., 2016; Ismaiel et al., 2016; Lu et al., 2016). However, MPTP-based PD models relies primarily on complex I inhibition but not on neuroinflammation itself (Bové and Perier, 2012; Machado et al., 2016). Hence, we have studied for the first time the effect of metformin in response to intranigral injection of LPS within the SN, a well-established and extensively used neuroinflammation-based model of PD (Herrera et al., 2005; Dutta et al., 2008). We analyzed the degree of microglia activation (OX-6 immunoreactivity) and polarization (pro-inflammatory/anti-inflammatory markers) and immunological checkpoints (CX3CR1 and CD200 expression). Our *in vivo* model using intranigral LPS revealed that metformin treatment decreased both the number of activated microglial cells and the acquisition of pro-inflammatory markers. Among the last ones, a remarkable effect of metformin in preventing LPS-induced IL-1β mRNA levels was found, thus further supporting our *in vitro* study showing that metformin highly inhibits NLRP3 inflammasome priming in microglia.

Under brain pathological conditions, microglial checkpoints pathways sense the environment to prevent overreaction to external stimuli. However, such checkpoint mechanisms may also limit the ability of microglia to protect the CNS (Deczkowska et al., 2018; Hickman et al., 2018). These include (a) CX3CR1–fractalkine sensing, which is highly relevant in homeostatic microglia (Keren-Shaul et al., 2017; Krasemann et al., 2017). Still, activation of CX3CR1 has been related to a decrease in the neurotoxic actions of microglia (Cardona et al., 2006); (b) CD200/CD200R, which attenuates microglia activation especially under inflammatory conditions (Barclay et al., 2002). CD200 is an extrinsic factor produced by neurons, astrocytes and oligodendrocytes; in the CNS, its receptor CD200R appears exclusively in microglia and macrophages (Hoek et al., 2000; Barclay et al., 2002; Biber et al., 2007). As expected, LPS injection downregulated the expression of both CD200 and CX3CR1 mRNAs in the ventral mesencephalon. Strikingly, metformin treatment full prevented the LPS-induced downregulation of CD200 and CX3CR1. To our knowledge, this is the first report pointing up metformin as a molecule responsible for inactivating microglia through checkpoints pathways.

Mitogen-activated protein kinases are a family of serine/threonine protein kinases that play important roles in signal transduction from a diverse array of extracellular stimuli, including LPS (Qin et al., 2007). This family of proteins is involved in the survival, proliferation, differentiation and apoptosis of nervous cells, therefore playing a role in neuroprotection/neurodegeneration (Kim and Choi, 2010). In previous works, we have demonstrated that LPS is able to increase the phosphorylation levels of JNK and p38 in different brain structures, and that these levels increase with the co-existence of other pro-inflammatory stimuli, such as chronic stress (Espinosa-Oliva et al., 2011). We thus studied the effect of metformin on the expression of key MAPKs after LPS treatment. Metformin treatment prevented the LPS-induced phosphorylation of JNK and p38 in LPS-injected rats. These results support the additional involvement of MAPKs signaling pathway in the immunosuppressive role of metformin in brain inflammation. Taken together, we provide evidence that metformin inhibits microglia reactivity through different mechanisms including MAPK signaling and checkpoints pathways thus reducing typical features of pro-inflammatory microglia including NADPH-dependent production of free radicals and NLRP3 inflammasome priming and activation.

Since the intranigral injection of LPS is a neuroinflammation-based model of PD (Castaño et al., 1998; Herrera et al., 2000), we analyzed the effect of metformin on the integrity of the nigrostriatal dopaminergic system. Unexpectedly, metformin treatment not only failed to protect the LPS-induced damage to the nigral dopaminergic system but exacerbated it. Our data support a metformin-associated inflammation-independent deleterious effect on the integrity of the nigrostriatal dopaminergic system. A distinguished feature of nigral dopaminergic neurons is the extremely complex axonal arborization that would greatly exceed those of other neuronal phenotypes and hence the associated energy demand (Pissadaki and Bolam, 2013; Hunn et al., 2015). Complex I deficiency has been long associated with mitochondrial dysfunction and PD risk (Schapira and Jenner, 2011). Interestingly, metformin is a weak inhibitor of complex I of the mitochondrial respiratory chain that may be critical under stressful conditions (El-Mir et al., 2000; Owen et al., 2000). In keeping with this view, we have demonstrated that metformin exacerbated dopaminergic damage in response to MPTP, a well-known complex I inhibitor (Ismaiel et al., 2016). The present study confirms and extends the view that metformin may be harmful to the dopaminergic system under different conditions including MPTP challenge or robust LPS-induced brain inflammation. Metformin is the first-choice treatment of diabetes mellitus II, a disease with high prevalence nowadays that seems to be a risk factor for some neurodegenerative diseases such as PD (Hu et al., 2007; Simon et al., 2007; Driver et al., 2008; D’Amelio et al., 2009; Miyake et al., 2010; Schernhammer et al., 2011; Xu et al., 2011). Only a few studies have addressed the effect of metformin on PD animal models with apparently conflicting effects, including beneficial (Patil et al., 2014; Lu et al., 2016; Katila et al., 2017) or detrimental (Chen et al., 2016; Ismaiel et al., 2016; Kuan et al., 2017; Thangthaeng et al., 2017). Although these apparent contradictory results may be due to differences in the experimental design, our study urges the need to carefully assess the use of metformin for the treatment of diabetes in patients who may suffer from PD.

## Author Contributions

KT and IG-D carried out the cell cultures, PCR, and WB. AI executed surgery, immunohistochemistry, and immunofluorescence techniques. AB-S and TD contributed with the cell culture. AE-O carried out the analysis and interpretation of data, and contributed to writing the manuscript. AM helped with the writing of the manuscript. AH contributed to statistical analysis and helped with the writing. JV contributed to experimental design and coordination, and helped with the writing. RdP conceived the study, contributed to experimental design and coordination, and helped with the writing.

## Conflict of Interest Statement

The authors declare that the research was conducted in the absence of any commercial or financial relationships that could be construed as a potential conflict of interest.

## Supplementary Material

FIGURE S1 Effect of metformin on Iba-1, IKKβ and arginase immunostaining in the SN after saline solution **(A–C,G–I)** and SalMetf **(D–F,J–L)** treatments. Iba-1 immunofluorescence in **(A,D)**, and IKKβ immunofluorescence in **(B,E)**, show a scarce induction of IKKβ in Iba-1-labeled microglial cells (merge, **C,F**). Arginase **(H,K)** is not induced in Iba-1-labeled microglial cells (**G,H,J,K**; merge **I,L**) in the animals treated with saline solution and SalMetf. Scale bar: 200 μm.

FIGURE S2 Effect of metformin and LPS on Iba-1, IKKβ and arginase immunostaining in the SN after LPS **(A–C,G–I)** and LPSMetf **(D–F,J–L)** treatments. Iba-1 immunofluorescence in **(A,D)**, and IKKβ immunofluorescence in **(B,E)** show an induction of IKKβ in Iba-1-labeled microglial cells (merge **C,F**). Arginase **(H,K)** is not induced in Iba-1-labeled microglial cells, except for a few ones (**G,H,J,K**; merge **I,L**) in the animals treated with LPS and LPSMetf. Images in the inserts in **(C,F,I,L)** are high-magnification photographs of the small white boxes in their respective panels. Scale bars: **(A–L)**, 200 μm; insert, 25 μm.

FIGURE S3 Photographs showing raw bands of IL-1b and GAPDH for the different treatments assayed (four independent experiments in each case). Treatments to BV2 cells: 1, none (control); 2, LPS; 3, ATP; 4, metformin; 5, LPS + metformin; 6, ATP + metformin; 7, LPS + ATP; 8, LPS + ATP + metformin.

FIGURE S4 Photographs showing raw bands of IL-1b and GAPDH for the different treatments assayed (three independent experiments in each case). Treatments to BV2 cells: 1, none (control); 2, LPS; 3, LPS + metformin; 4, ATP; 5, LPS + ATP; 6, LPS + ATP + metformin; 7, Metformin; 8, Metformin + ATP.

FIGURE S5 Photographs showing raw bands of GAPDH, JNK, P-JNK, p38, and P-p38 for the different treatments assayed (four independent experiments in each case). Sal, animals injected with saline solution in the SN plus oral administration of water; SalMetf, animals injected with saline solution in the SN plus oral administration of metformin; LPS, animals injected with LPS in the SN plus oral administration of water; LPSMetf, animals injected with LPS in the SN plus oral administration of metformin.

FIGURE S6 Photographs showing raw bands of JNK, P-JNK, and GAPDH for the different treatments assayed (four independent experiments in each case). Treatments: 1 and 5, Sal, animals injected with saline solution in the SN plus oral administration of water; 2 and 6, SalMetf, animals injected with saline solution in the SN plus oral administration of metformin; 3 and 7, LPS, animals injected with LPS in the SN plus oral administration of water; 4 and 8, LPSMetf, animals injected with LPS in the SN plus oral administration of metformin.

## Abbreviations

CX3CR1: CX3C chemokine receptor 1
ELISA: enzyme-linked immunosorbent assay
GFAP: glial fibrillary acidic protein
IKKβ: IκB kinase β
IL: interleukin
iNOS: inducible nitric oxide synthase
LPS: lipopolysaccharide
MAPK: mitogen-activated protein kinase
MCP-1: Monocyte chemoattractant protein 1
MPTP: 1-methyl-4-phenyl-1,2,3,6-tetrahydropyridine
NLRP3: nucleotide-binding oligomerization domain-like receptor protein containing a pyrin domain 3
PBS: phosphate-buffered saline
PD: Parkinson’s disease
ROS: reactive oxygen species
SN: substantia nigra
TBS: Tris-buffered saline
TBST: TBS Tween
TH: tyrosine hydroxylase
TNF: tumor necrosis factor
