# Supplementary figures and images for: Divergent Effects of Metformin on an Inflammatory Model of Parkinson’s Disease
Source: Front Cell Neurosci. 2018 Nov 21;12:440. doi: 10.3389/fncel.2018.00440 (PMC6258993; doi:10.3389/fncel.2018.00440)

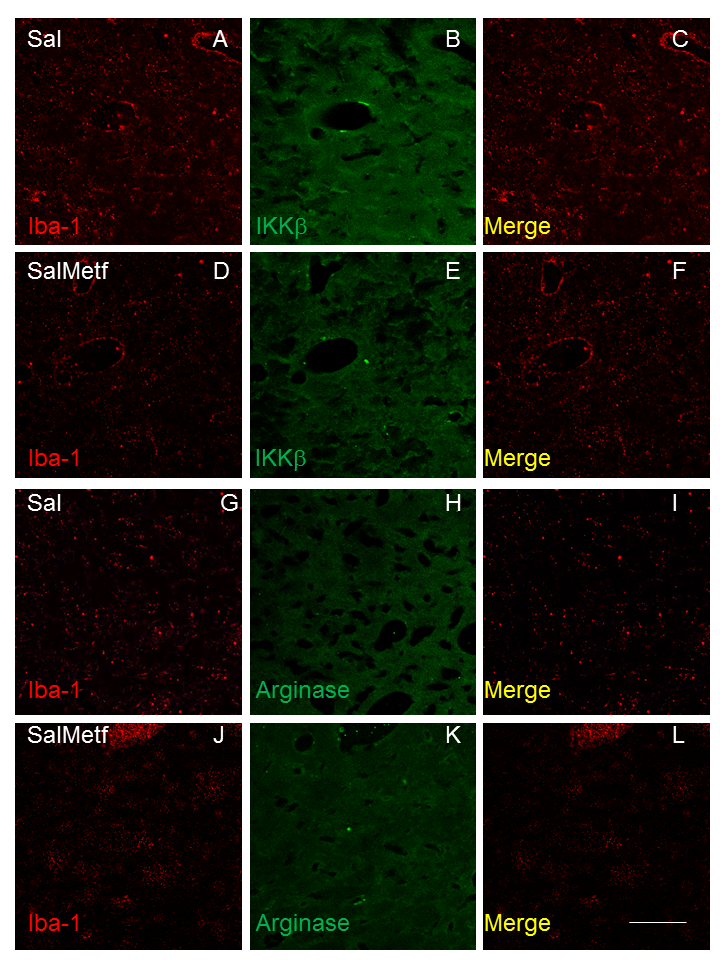

Supplement: FIGURE S1 — Effect of metformin on Iba-1, IKKβ and arginase immunostaining in the SN after saline solution (A–C,G–I) and SalMetf (D–F,J–L) treatments. Iba-1 immunofluorescence in (A,D), and IKKβ immunofluorescence in (B,E), show a scarce induction of IKKβ in Iba-1-labeled microglial cells (merge, C,F). Arginase (H,K) is not induced in Iba-1-labeled microglial cells (G,H,J,K; merge I,L) in the animals treated with saline solution and SalMetf. Scale bar: 200 μm. [file Image_1.TIF]

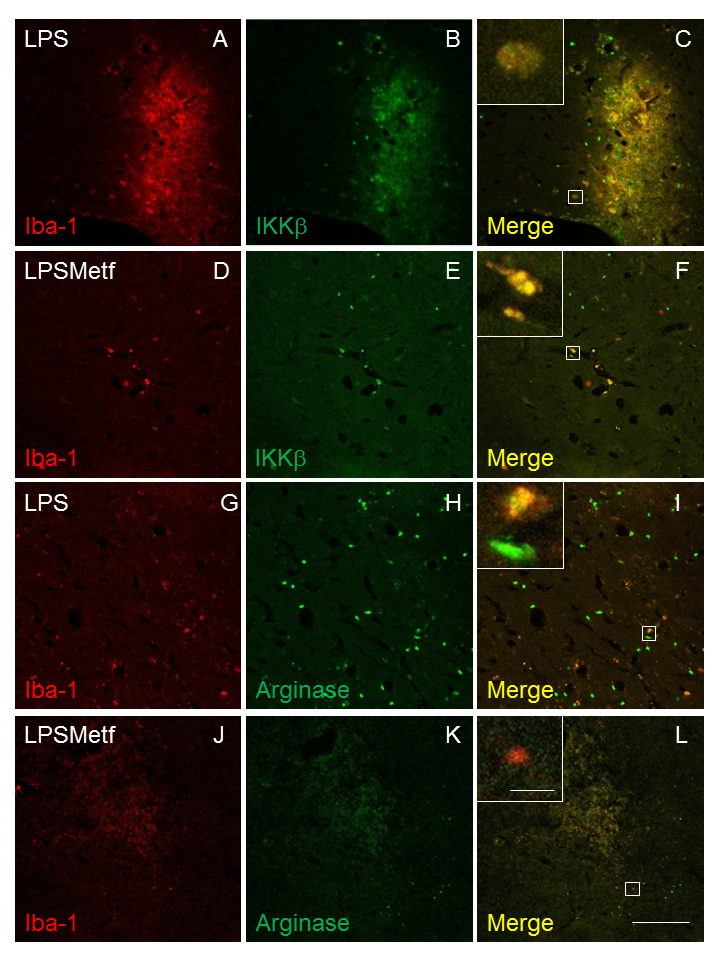

Supplement: FIGURE S2 — Effect of metformin and LPS on Iba-1, IKKβ and arginase immunostaining in the SN after LPS (A–C,G–I) and LPSMetf (D–F,J–L) treatments. Iba-1 immunofluorescence in (A,D), and IKKβ immunofluorescence in (B,E) show an induction of IKKβ in Iba-1-labeled microglial cells (merge C,F). Arginase (H,K) is not induced in Iba-1-labeled microglial cells, except for a few ones (G,H,J,K; merge I,L) in the animals treated with LPS and LPSMetf. Images in the inserts in (C,F,I,L) are high-magnification photographs of the small white boxes in their respective panels. Scale bars: (A–L), 200 μm; insert, 25 μm. [file Image_2.TIF]

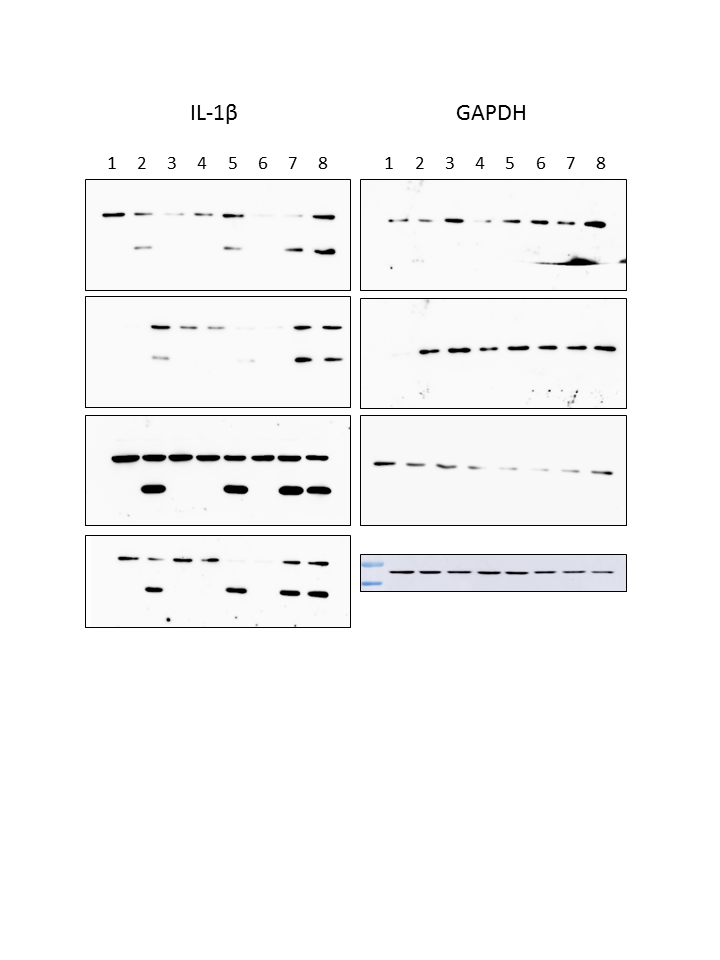

Supplement: FIGURE S3 — Photographs showing raw bands of IL-1b and GAPDH for the different treatments assayed (four independent experiments in each case). Treatments to BV2 cells: 1, none (control); 2, LPS; 3, ATP; 4, metformin; 5, LPS + metformin; 6, ATP + metformin; 7, LPS + ATP; 8, LPS + ATP + metformin. [file Image_3.TIF]

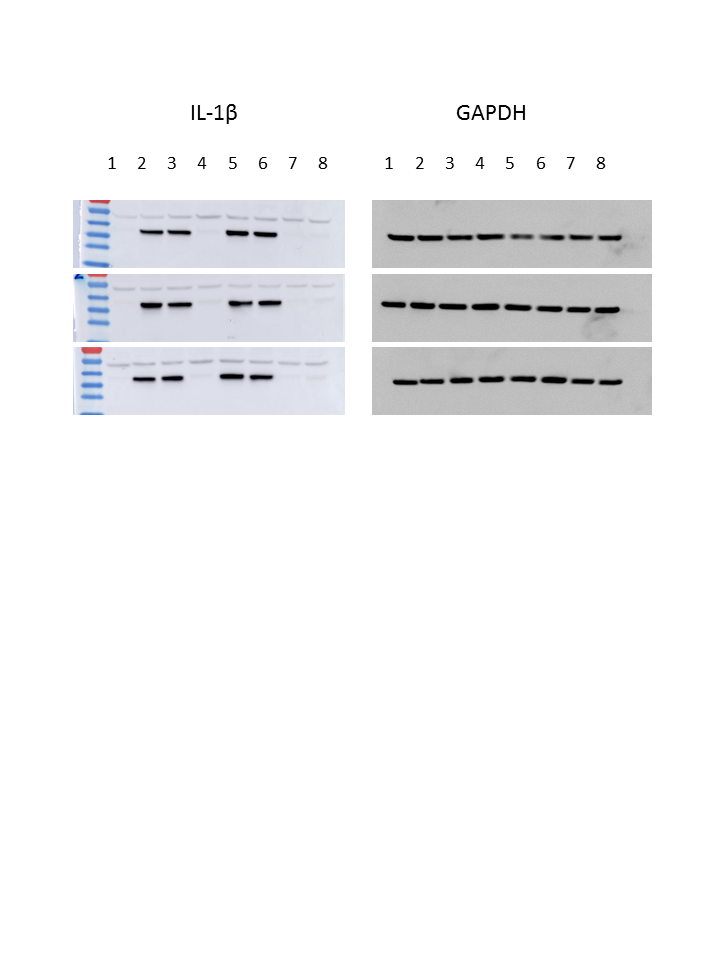

Supplement: FIGURE S4 — Photographs showing raw bands of IL-1b and GAPDH for the different treatments assayed (three independent experiments in each case). Treatments to BV2 cells: 1, none (control); 2, LPS; 3, LPS + metformin; 4, ATP; 5, LPS + ATP; 6, LPS + ATP + metformin; 7, Metformin; 8, Metformin + ATP. [file Image_4.TIF]

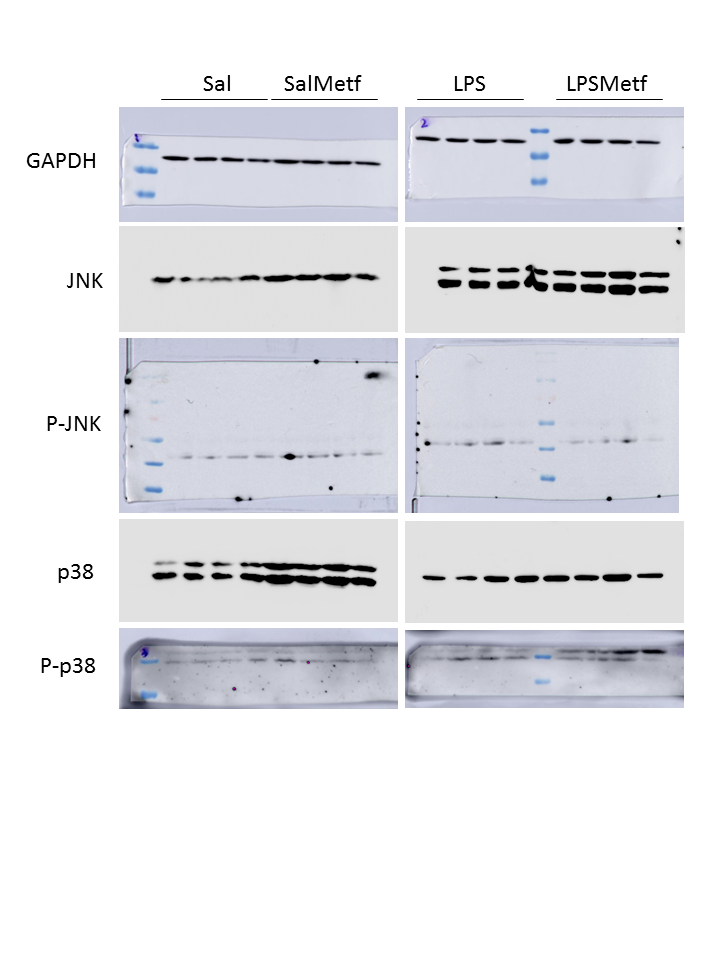

Supplement: FIGURE S5 — Photographs showing raw bands of GAPDH, JNK, P-JNK, p38, and P-p38 for the different treatments assayed (four independent experiments in each case). Sal, animals injected with saline solution in the SN plus oral administration of water; SalMetf, animals injected with saline solution in the SN plus oral administration of metformin; LPS, animals injected with LPS in the SN plus oral administration of water; LPSMetf, animals injected with LPS in the SN plus oral administration of metformin. [file Image_5.TIF]

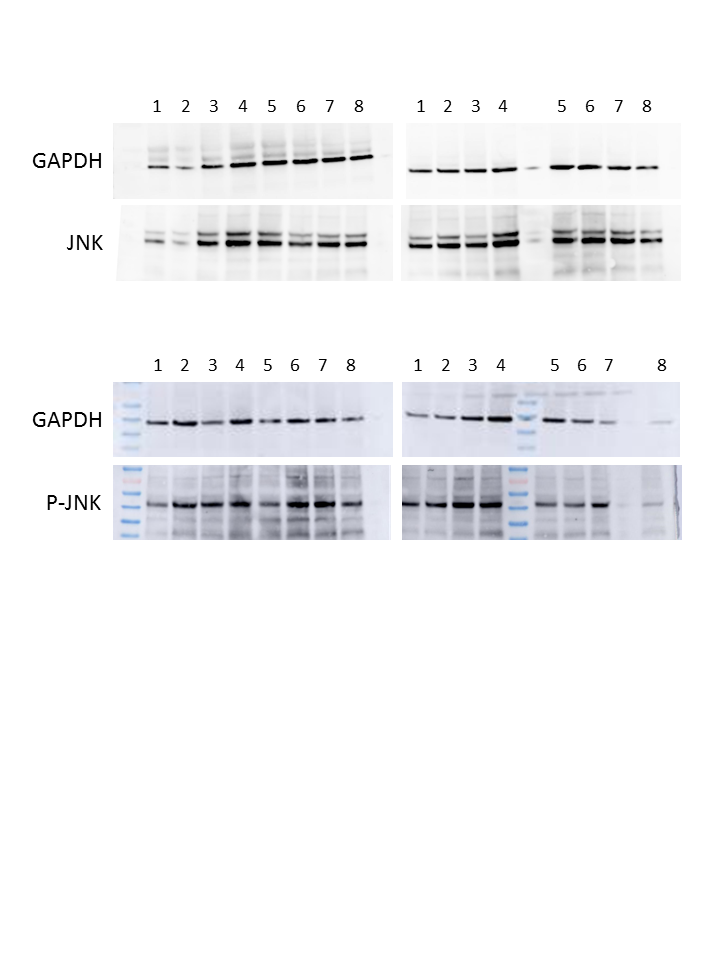

Supplement: FIGURE S6 — Photographs showing raw bands of JNK, P-JNK, and GAPDH for the different treatments assayed (four independent experiments in each case). Treatments: 1 and 5, Sal, animals injected with saline solution in the SN plus oral administration of water; 2 and 6, SalMetf, animals injected with saline solution in the SN plus oral administration of metformin; 3 and 7, LPS, animals injected with LPS in the SN plus oral administration of water; 4 and 8, LPSMetf, animals injected with LPS in the SN plus oral administration of metformin. [file Image_6.TIF]
